# Prevalence and Risk Factors for Gastroesophageal Reflux Disease (GERD) Among Visitors to the Health Center of Imam Mohammad Ibn Saud Islamic University

**DOI:** 10.7759/cureus.43936

**Published:** 2023-08-22

**Authors:** Khalid I AlHussaini, Fahad B Bin Abbas, Shawq F Aljabri, Rawan A Bayamin, Yara A Alfraih, Somiah A Alsarar

**Affiliations:** 1 Department of Internal Medicine, Imam Mohammad Ibn Saud Islamic University (IMSIU), Riyadh, SAU; 2 Department of Internal Medicine, King Faisal Specialist Hospital and Research Centre, Riyadh, SAU; 3 College of Medicine, Imam Mohammad Ibn Saud Islamic University (IMSIU), Riyadh, SAU

**Keywords:** healthcare center, prevalence gerd, risk factors gerd, gerd patients, gerd

## Abstract

Background: A considerable majority of people have gastroesophageal reflux disease (GERD), a common gastrointestinal ailment. Globally, the prevalence of GERD has been rising, and it is linked to several risk factors. In this study, the incidence of GERD in a sample of the population was examined, along with the associated factors that may have an impact on it.

Methodology: The Gastroesophageal Reflux Disease Questionnaire (GERD-Q) was included in a self-administered survey given to 490 participants in a cross-sectional study to help determine who was more likely to have GERD. The questionnaire collected data on demographic elements, health-related traits, and past GERD diagnoses.

Results: The findings revealed that 32.7% of the individuals had previously received a GERD diagnosis. Of the patients, 17.1% had a GERD-Q score of 8 or above, which indicates a higher likelihood of having GERD. Participants who had previously been diagnosed with GERD had a noticeably greater incidence of GERD, and females had a higher incidence of GERD than males. The frequency of caffeine consumption was substantially correlated with the occurrence of GERD.

Conclusion: Our study emphasizes the value of early GERD diagnosis and therapy to reduce problems and enhance the quality of life for those who are affected. According to our research, coffee use, gender, and prior GERD diagnoses are all linked to an increased risk of developing GERD. The GERD-Q is a trustworthy and proven tool for GERD diagnosis and might be used in clinical practice to recognize GERD patients and offer suitable treatment. Additional research is required to determine how additional risk variables affect the prevalence of GERD.

## Introduction

Gastroesophageal reflux disease (GERD) is plausibly one of the most prevalent diseases seen by a gastroenterologist. GERD is described as symptoms or complications caused by the reflux of stomach acid and contents into the esophagus or beyond, into the oral cavity (including the larynx), or the lung [1,2]. Various factors can influence the development of GERD; some of them are non-modifiable such as age, gender, family history, and obesity, while others are modifiable such as consumption of certain types of food or drinks, limited physical activity, consuming non-steroidal anti-inflammatory drugs (NSAIDs), and smoking [3-5]. Different studies done in Europe, America, and the Middle East population found the prevalence rates ranged from 10% to 30% while the rates in East Asia were lower than 10% [4,6,7]. However, few studies have been performed in Saudi Arabia to estimate the prevalence of GERD. We aim to study the prevalence and risk factors for GERD among visitors to the health center of Imam Mohammad Ibn Saud Islamic University. In the population of Saudi Arabia, the prevalence of GERD varies between 28.7% and 45.4%, based on two different studies [8,9]. On the other hand, obesity, defined according to the World Health Organization's classification as a body mass index (BMI) of 30 kg/m2, is associated with a reduced health-related quality of life and several chronic diseases. 

## Materials and methods

This cross-sectional study was conducted at Imam Mohammad Ibn Saud Islamic University (IMSIU) medical center, Riyadh, Saudi Arabia. The sample size was approximately 500 participants as estimated by the OpenEpi web tool, with a 95% confidence level. The prevalence used to calculate this sample size was 9.4%. The study included all nationalities of either sex who understood Arabic or English. Participants who were aged 17 or older and agreed to participate in this study and visited IMSIU medical center for any reason were included. Participants who did not complete the questionnaire and who are not residents of the central region in Saudi Arabia and are not visitors of the IMSIU medical center were excluded. The Institutional Review Board (IRB) of the IMSIU medical center reviewed and issued approval (No. 353/2022).

Data collection

Data was collected using face-to-face interviews to ensure the accuracy of data collection in the medical center for all participants fulfilling the inclusion criteria. A multi-item questionnaire was used, which was adopted from a previously published study that used the Gastroesophageal Reflux Disease Questionnaire (GERD-Q) [10]. The questionnaire consists of questions that depend on the type and frequency of symptoms experienced by respondents. The respondents who get a score of 8 or more have a higher possibility of having GERD, while those who get <8 have a low or no possibility of having GERD. The GERD questionnaire has a sensitivity of 65% and a specificity of 71% for GERD diagnosis. The second part includes questions about the risk factors of GERD, such as BMI, coffee, tea, and chocolate ingestion, energetic drinks, soft drinks, fried food, sports activities, smoking, and family history. Further, we measured participants’ height and weight to determine their BMI. 

Data management and analysis plan

After collecting the subjects that met our criteria, the obtained information was analyzed via IBM SPSS Statistics for Windows, Version 26.0 (Armonk, NY: IBM Corp) with the help of an expert in data analysis. Frequency and percentage were used for the description of categorical variables, while mean and standard deviation were used for describing the ongoing variables. Chi-square, ANOVA, and t-tests were used to assess the relationship between demographic and health factors and the incidence of GERD. All statements were considered significant when the p-value was lower than 0.05.

## Results

The overall number of participants in this study was 490 with a 99.4% response rate, with 201 (41.0%) men and 289 (59.0%) women and most of the participants (62.7%) were over the age of 27. The average weight was 73.09 kg, the average height was 163.88 cm, and the average BMI was 27.15 kg/m^2^ (SD: 8.94). Of the study participants, 62% were either overweight or obese, making up the majority of the group. Of the study participants, 32.7% (n=160) said they had previously received a GERD diagnosis. Of those, 114 (71.3%) were diagnosed based on clinical symptoms, seven (4.4%) were identified based on an esophageal acid test, and 39 (24.4%) were diagnosed based on a gastrointestinal endoscopy (Table 1).

**Table 1:** Demographic factors of the participants (N=490)

		Count	Column N %
Gender	Male	201	41.0%
	Female	289	59.0%
Age	18-20	53	10.8%
	21-23	75	15.3%
	24-26	55	11.2%
	27 or older	307	62.7%
Height (cm)	Mean (SD)	163.88 (8.99)	
Weight (kg)	Mean (SD)	73.09 (17.77)	
BMI (kg/m^2^)	Mean (SD)	27.15 (8.94)	
BMI category	Underweight	20	4.1%
	Normal weight	166	33.9%
	Overweight	182	37.1%
	Obese	122	24.9%
Had you diagnosed with GERD before?	No	330	67.3%
	Yes	160	32.7%
How you had been diagnosed?	Based on symptoms at clinic	114	71.3%
	Esophageal acid test	7	4.4%
	Endoscopy of the gastrointestinal tract	39	24.4%

The participant's health-related factors are shown in Table 2. Of the study participants, 87.1% (n=427) reported not smoking, 10.4% (n=51) reported smoking now, and 2.6% (n=12) claimed to be former smokers. The mean number of years that current and former smokers smoked was 10.94 (SD=8.94). Of the study participants, 73.9% (n=362) said they drank caffeine, 25.4% (n=92) said they drank one cup, 32.6% (n=118) said they drank two cups, 18.8% (n=68) said they drank three cups, and 23.2% (n=84) said they drank more than three cups per day. The majority of individuals (65.1%, n=319) admitted to having poor eating habits such as eating unhealthy food and eating spicy food (62.7%, n=307). In addition, 68.4% (n=335) reported eating within three hours of going to bed, and 28.4% (n=139) reported eating a lot of food.

**Table 2:** Health-related factors of the participants

		Count	Column N %
Smoking	No	427	87.1%
	Yes	51	10.4%
	Ex-smoking	12	2.6%
Years of smoking	Mean (SD)	10.94 (8.94)	
Caffeine consumption	No	128	26.1%
	Yes	362	73.9%
Number of consumed caffeinated drinks during the day	One cup	92	25.4%
	Two cups	118	32.6%
	Three cups	68	18.8%
	More than 3 cups	84	23.2%
Do you have bad eating habits?	No	171	34.9%
	Yes	319	65.1%
Do you eat spicy food?	No	183	37.3%
	Yes	307	62.7%
Do you eat large amounts of food?	No	351	71.6%
	Yes	139	28.4%
Do you eat in the 3 hours before bed?	No	155	31.6%
	Yes	335	68.4%

**Figure 1:**
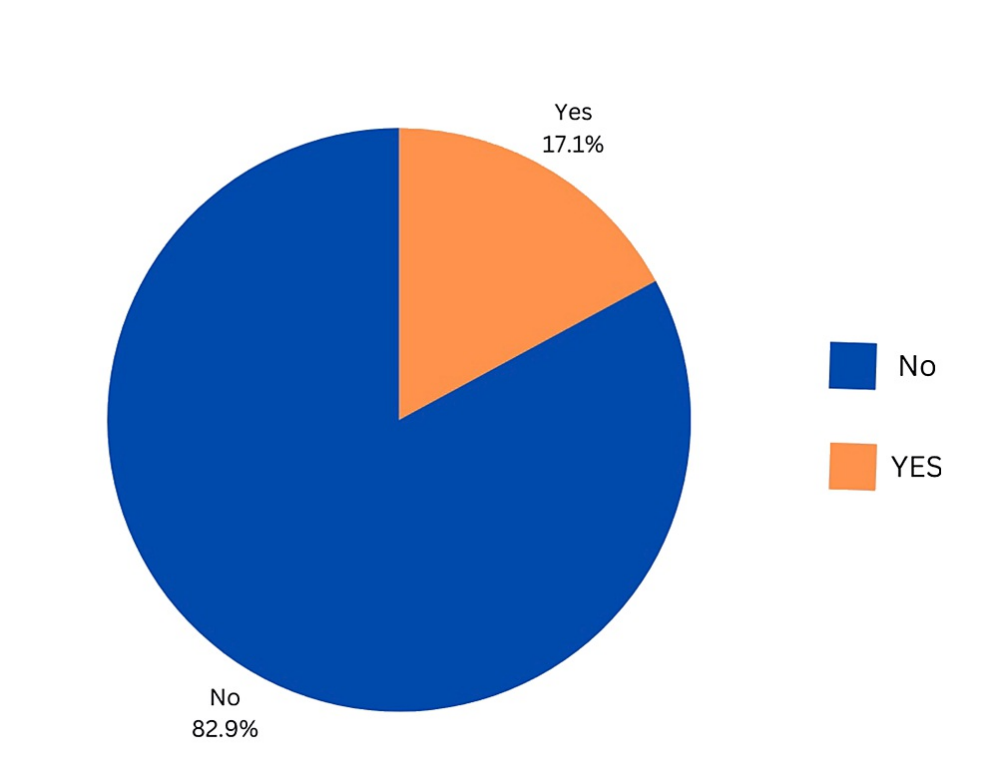
Prevalence of GERD according to the Gastroesophageal Reflux Disease Questionnaire (GERD-Q) GERD: gastroesophageal reflux disease

Figure 1 depicts the prevalence of GERD. Of the 490 participants, 84 (17.1%) had a GERD-Q score of 8 or above, which indicates an increase in the likelihood of having GERD. However, 406 (82.9%) of the participants had a score of less than 8, indicating little to no chance of getting GERD.

The correlation between GERD incidence and participant demographics is seen in Table 3. Gender and GERD incidence were significantly correlated (p-value=0.011), with a larger percentage of females than males having the condition (20.8% compared with 11.9%). Age and BMI category did not significantly correlate with the prevalence of GERD (p-values: 0.152 and 0.101, respectively). However, participants who had already received a GERD diagnosis had a considerably higher incidence of the condition (41.9%, n=67) than participants who had not (5.2%, n=17) (p-value=0.000).

**Table 3:** Relationship between GERD incidence and demographic factors of the participants GERD: gastroesophageal reflux disease

		Diagnosis with GERD				
		No		Yes		P-value
		Count	Row N %	Count	Row N %	
Gender	Male	177	88.1%	24	11.9%	0.011
	Female	229	79.2%	60	20.8%	
Age	18-20	50	94.3%	3	5.7%	0.101
	21-23	61	81.3%	14	18.7%	
	24-26	47	85.5%	8	14.5%	
	27 or older	248	80.8%	59	19.2%	
BMI category	Underweight	17	85.0%	3	15.0%	0.152
	Normal weight	143	86.1%	23	13.9%	
	Overweight	153	84.1%	29	15.9%	
	Obese	93	76.2%	29	23.8%	
Had you diagnosed with GERD before?	No	313	94.8%	17	5.2%	0.000
	Yes	93	58.1%	67	41.9%	

The association between GERD incidence and participant health-related factors is shown in Table 4. Smoking status, caffeine consumption, poor eating habits, eating spicy food, eating a lot of food, and eating three hours before bed did not significantly affect the risk of GERD (p-values = 0.337, 0.575, 0.863, 0.279, 0.755, and 0.833, respectively). However, a higher percentage of participants who consumed two or more caffeinated drinks per day were diagnosed with GERD than those who drank one or no caffeinated drinks per day (p-value=0.040). 

**Table 4:** Relationship between GERD incidence and health-related characteristics of the participants GERD: gastroesophageal reflux disease

		Diagnosis with GERD				
		No		Yes		P-value
		Count	Row N %	Count	Row N %	
Smoking	No	350	82.0%	77	18.0%	0.337
	Yes	46	90.2%	5	9.8%	
	Ex-smoking	10	83.3%	2	16.7%	
Caffeine consumption	No	104	81.3%	24	18.8%	0.575
	Yes	302	83.4%	60	16.6%	
Number of consumed caffeinated drinks during the day	One cup	85	92.4%	7	7.6%	0.040
	Two cups	95	80.5%	23	19.5%	
	Three cups	57	83.8%	11	16.2%	
	More than 3 cups	65	77.4%	19	22.6%	
Do you have bad eating habits?	No	141	82.5%	30	17.5%	0.863
	Yes	265	83.1%	54	16.9%	
Do you eat spicy food?	No	156	85.2%	27	14.8%	0.279
	Yes	250	81.4%	57	18.6%	
Do you eat large amounts of food?	No	292	83.2%	59	16.8%	0.755
	Yes	114	82.0%	25	18.0%	
Do you eat in the 3 hours before bed?	No	129	83.2%	26	16.8%	0.833
	Yes	277	82.7%	58	17.3%	

## Discussion

A considerable majority of people have GERD, a common gastrointestinal ailment [2,11,12]. GERD is more common than it used to be, with a reported frequency of 10% to 20% in Western nations [13]. Risk factors for GERD include smoking, hiatal hernias, obesity, and particular dietary practices. In this study, we sought to determine the prevalence of GERD diagnoses and the contributing factors that might have an impact on GERD incidence in a sample of the general population. Our findings showed that 32.7% of the individuals had previously received a GERD diagnosis. This incidence is consistent with earlier investigations, which found that diagnosed GERD was prevalent in the general community including the study of Al Ghadeer et al. in the eastern region of Saudi Arabia and the study of Almadi et al. which showed that the prevalence of GERD among Saudi residents ranged between 23.47% and 45.4% [4,9]. The prevalence of GERD may be higher, it is important to note, as many sufferers do not seek medical attention or do not receive a diagnosis. In addition, to find people who had a higher chance of developing GERD, we employed the GERD-Q. Our findings revealed that 17.1% of the participants had a GERD-Q score of 8 or above, indicating a greater likelihood of having GERD. Our results were lower than those reported by different studies conducted in Saudi Arabia using GERD-Q where Alsuwat et al. showed a prevalence of 28.7% among the 2,043 Saudi general population [8], the study of Kariri et al. which reported a prevalence of 32.2% among the general population in southwestern Saudi Arabia [13] while in Makkah, Halawani and Banoon showed that GERD frequency rate was 17.4% among the participants [3]. Additionally, significantly higher incidence rates were also noted; Alsulobi et al. reported rates as high as 61.8% [14]. Altwigry et al. limited their group to only Saudi teachers in the same situation and reported a prevalence rate of 55% [15]. The reported rates were different for a variety of reasons, including the various instruments utilized for patient assessments and the unique personal and environmental circumstances of each patient. Despite using the GERD-Q evaluation approach, Al-Humayed et al. report a 15% ascendance rate in the southern part of Saudi Arabia in an endoscopy-based assessment more comparable to our estimated prevalence rate [16]. GERD-Q is a trustworthy and approved tool for GERD diagnosis [17,18]. The findings of our study demonstrate that the GERD-Q can reliably identify people who have GERD because participants who had previously received a GERD diagnosis had a considerably greater incidence of GERD than participants who had not. According to the results of our investigation, the incidence of GERD was substantially correlated with gender. In comparison to men, women had a higher incidence of GERD. Given the prevalence of GERD, no gender differences were reported to be significant in several earlier investigations [8,13,14,19], which is inconsistent with our findings. Alrashed et al.'s findings [12] indicating the male population was more susceptible to developing GERD were corroborated by a study from India that made comparable discoveries [20]. According to various epidemiological studies, which support our findings, men's esophageal mucosal epithelium is more vulnerable to the reflux of gastroduodenal contents than women's, even though women are more likely to experience GERD symptoms [21-24]. Estrogen has been demonstrated to enhance the risk of GERD, hence hormonal reasons may be to blame for this gender disparity in GERD incidence [21,25,26]. Age or BMI category did not significantly correlate with the occurrence of GERD, according to our study. At first, we did not detect any age-related statistical significance. Kariri et al. [13] was the only Saudi study to discover significance according to age, and two more non-Saudi investigations [27,28] corroborated their findings. We discovered no significance, which is in line with the findings of Alrashed et al. [12], Alsuwat et al. [8], and Alsulobi et al. [14]. Being overweight or obese was not shown to be a habitual risk factor, which was consistent with the findings of Kariri et al. [13], who likewise reported no relevance. On the other hand, Alkhathami et al. [29] found that obesity increases the chance of developing GERD in their study. However, the number of caffeinated drinks ingested each day was strongly related to the occurrence of GERD, with a higher proportion of participants who drank two or more caffeinated drinks per day being diagnosed with the condition than those who drank one or none. There was no correlation between coffee consumption and GERD according to the data from the meta-analysis by Kim et al., which included 15 case-control studies [30]. The incidence of GERD varies across groups and geographical areas, and it's crucial to remember that GERD-Q identification and diagnosed GERD may not accurately reflect the true frequency of GERD in the population. Additional research is required to determine the frequency of GERD in various groups and the factors that contribute to its occurrence. To avoid complications from GERD and enhance the quality of life for those who are affected, our study emphasizes the value of early diagnosis and treatment. According to our research, coffee use, gender, and prior GERD diagnoses are all linked to an increased risk of developing GERD. The GERD-Q is a trustworthy and proven tool for GERD diagnosis and might be used in clinical practice to recognize GERD patients and offer suitable treatment. Additional research is required to determine how dietary practices and lifestyle choices, among other risk factors, affect the occurrence of GERD.

## Conclusions

Our study emphasizes the value of early GERD diagnosis and therapy to reduce problems and enhance the quality of life for those who are affected. According to our research the prevalence of GERD is 17.1%. Coffee use, gender, and prior GERD diagnoses are all linked to an increased risk of developing GERD. Additional research is required to determine how additional risk variables affect the prevalence of GERD.
